# Patch dynamics modeling framework from pathogens’ perspective: Unified and standardized approach for complicated epidemic systems

**DOI:** 10.1371/journal.pone.0238186

**Published:** 2020-10-15

**Authors:** Shi Chen, Yakubu Owolabi, Ang Li, Eugenia Lo, Patrick Robinson, Daniel Janies, Chihoon Lee, Michael Dulin

**Affiliations:** 1 Department of Public Health Sciences, University of North Carolina Charlotte, Charlotte, NC, United States of America; 2 School of Data Science, University of North Carolina Charlotte, Charlotte, NC, United States of America; 3 Division of HIV and TB, Centers for Disease Control and Prevention, Atlanta, GA, United States of America; 4 State Key Laboratory of Vegetation and Environmental Change, Chinese Academy of Sciences, Beijing, China; 5 Department of Biological Sciences, University of North Carolina Charlotte, Charlotte, NC, United States of America; 6 Academy of Population Health Innovation, University of North Carolina Charlotte, Charlotte, NC, United States of America; 7 Department of Bioinformatics, University of North Carolina Charlotte, Charlotte, NC, United States of America; 8 School of Business, Stevens Institute of Technology, Hoboken, NJ, United States of America; University of Chester, UNITED KINGDOM

## Abstract

Mathematical models are powerful tools to investigate, simulate, and evaluate potential interventions for infectious diseases dynamics. Much effort has focused on the Susceptible-Infected-Recovered (SIR)-type compartment models. These models consider host populations and measure change of each compartment. In this study, we propose an alternative patch dynamic modeling framework from pathogens’ perspective. Each patch, the basic module of this modeling framework, has four standard mechanisms of pathogen population size change: birth (replication), death, inflow, and outflow. This framework naturally distinguishes between-host transmission process (inflow and outflow) and within-host infection process (replication) during the entire transmission-infection cycle. We demonstrate that the SIR-type model is actually a special cross-sectional and discretized case of our patch dynamics model in pathogens’ viewpoint. In addition, this patch dynamics modeling framework is also an agent-based model from hosts’ perspective by incorporating individual host’s specific traits. We provide an operational standard to formulate this modular-designed patch dynamics model. Model parameterization is feasible with a wide range of sources, including genomics data, surveillance data, electronic health record, and from other emerging technologies such as multiomics. We then provide two proof-of-concept case studies to tackle some of the existing challenges of SIR-type models: sexually transmitted disease and healthcare acquired infections. This patch dynamics modeling framework not only provides theoretical explanations to known phenomena, but also generates novel insights of disease dynamics from a more holistic viewpoint. It is also able to simulate and handle more complicated scenarios across biological scales such as the current COVID-19 pandemic.

## Introduction

Host-oriented population level compartment models for investigating infectious diseases dynamics, such as the commonly used Kermack-McKendrick model (also known as Ross model), were developed around 1920s [1–7], almost a century ago when diagnosis was largely relying on superficial symptoms of patients (hosts). With the rapid advancement of modern medicine, clinicians now not only know whether the potential patient has certain pathogenic microorganisms (presence-absence) through microbiology tests, but in many scenarios, they can quantify the actual pathogen load (e.g., viral load, number of copies of viral DNA/RNA in viral infections) via modern molecular techniques. While the widely used compartment models have made a tremendous amount of contributions to characterize and understand infectious diseases dynamics [8–10], there are quite a few major challenges [11–23]. Pathogen, host, and environment (sometimes linked through one or more vector species) together form an inseparable epidemiology triad, but the compartment models focus only on host populations. Therefore, pathogens, the actual cause of infectious diseases, are only modeled implicitly [16]. Individuals of the host population are usually categorized in discrete epidemiological states, such as susceptible (*S*), infected (*I*), and recovered (*R*), thus the name SIR-type models. For consistency of terminology, we call such host-oriented population level models as SIR-type models throughout this paper. This discretization of epidemiological states was reasonable a century ago when only clinical symptoms and signs rather than confirmatory laboratory data were available. However, such discretization is too coarse compared to the clinical information we can acquire now, e.g., viral load, colony forming units, and genomic sequence of specific pathogen strain. For example, a patient carrying 100 viral load and the other carrying 1000 viral load of a specific virus are both categorized as “*I*” state, nevertheless, the two patients differ substantially on potential disease transmissibility. To address this issue, current SIR-type models may have many more sub-divided compartments.

In addition, SIR-type modeling paradigm was originally tailored for human host population without consideration of possible future extensions. Therefore, it is technically difficult to incorporate environments and vectors later into the model. Nevertheless, non-host reservoirs play a critical role in many important infectious diseases, e.g., foodborne and waterborne diseases, healthcare associated infections (HAIs) through contaminated surfaces and devices [24, 25]. Environment is one of three major components of the epidemiological triad [26], a fundamental concept in infectious disease epidemiology (the other two being pathogen and host). Unfortunately, there is no consistent way to accurately quantify the role of the environment in the compartment model. It is unclear whether the environment needs to be modeled as a binary, density, ordinal, or nominal quantity.

Another intrinsic flaw of SIR-type models is unable to differentiate between-host transmission (e.g., pathogen transmitting from a vector, a contaminated environment, or another host) and within-host infection (i.e., pathogens entering host's body, replicating, and causing clinical symptoms) processes [13, 15]. In an SIR-type model, a single transmission coefficient (usually denoted as *β*) quantifies the combined effect of contact rate between susceptible (*S*) and infected (*I*) hosts (between-host), and per-contact probability of infection (within-host) [16]. Nevertheless, between-host transmission and within-host infection comprise a series of complicated and interdependent pathological, physiological, and immunological processes [12, 13]. In addition, simple SIR-type mode rely on the implicit assumption of homogeneous mixing. Therefore, we suggest that one single transmission coefficient is over-simplified and does not reflect the reality of the underlying transmission-infection processes.

Current state-of-the-art mathematical modeling work has recognized the theoretical and technical challenges and various cross-scale models (also known as multi-scale models) have been proposed to link both within- and between-host transmission [27–30]. These models are also able to track evolution of pathogens and resistance as well [30, 31], which is not feasible by the commonly used host-level SIR-type model. While these cross-scale models are pioneers in providing a more comprehensive characterizations of infectious disease dynamics, there are still some obstacles. First, most of these models focus on the host aspect and usually do not consider the environmental reservoir as a critical component. Second, there is still a lack of standardization and consistency of cross-scale model formulation, leading to inconsistency and lack of interoperability among these frameworks. Therefore, it is difficult to compare and evaluate different types of models. Third, pathogen demography such as pathogen replication and decay occur at different temporal resolution from human hosts, making cross-scale modeling difficult to incorporate both temporal resolutions.

To address these challenges, we propose an alternative modeling perspective and framework that focus on pathogens directly [32, 33]. Pathogens are, in fact, microorganisms in their own ecological niche [34, 35]. Like all microorganisms, pathogens come and go, pathogens are born and die in their ambient dwellings (patches). Environment, host, and vector are all pathogens’ temporary or persistent patch. Therefore, pathogens’ population dynamics can be modeled in a standardized way among different patches (human host, vector host, reservoir host, environment). On the other hand, because each host and/or environment is specifically modeled as their respective patch, this modeling framework is also an agent-based model (ABM) from host’s perspective. Our modeling framework can be formulated for interoperability using the existing “Overview, Design concepts, and Details” (ODD) protocol [36, 37]. In addition, since our modeling framework is standardized across pathogen patches, it avoids the potential pitfall of temporal resolution inconsistency in other commonly used cross-scale models [38].

The overarching goal of this study is to present a unified, standardized, and modular-designed mathematical modeling framework that explicitly tracks spatio-temporal dynamics of pathogen population between hosts, environments, and potential vectors. This pathogen patch dynamics model is based on rigorous ecological and epidemiological foundations while still relating to the existing SIR-type modeling paradigm. We provide standards of formulating the model, and demonstrate its feasibility and flexibility across scales with case studies as proof-of-concept to address some major challenges in infectious disease modeling field.

## Methods

### Generalized modeling framework from pathogen’s perspective

In this study, we establish a pathogen-focused infectious disease dynamics modeling framework other than the well-established paradigm of host-level SIR-type models. In our modeling framework, various hosts, environment, and vectors are all considered as temporary or persistent patches of pathogens, where pathogens can move in, move out, be born, and die. Between-host (either direct host-to-host or indirect environment-to host and vector-to-host) transmission is described as pathogen (sub)population movement from its original patch (host, environment, or host) *i* to destiny patch (host) *j*. Within-host infection is described as successful pathogen replication within the host, which usually results in clinical symptoms.

**Fig 1:**
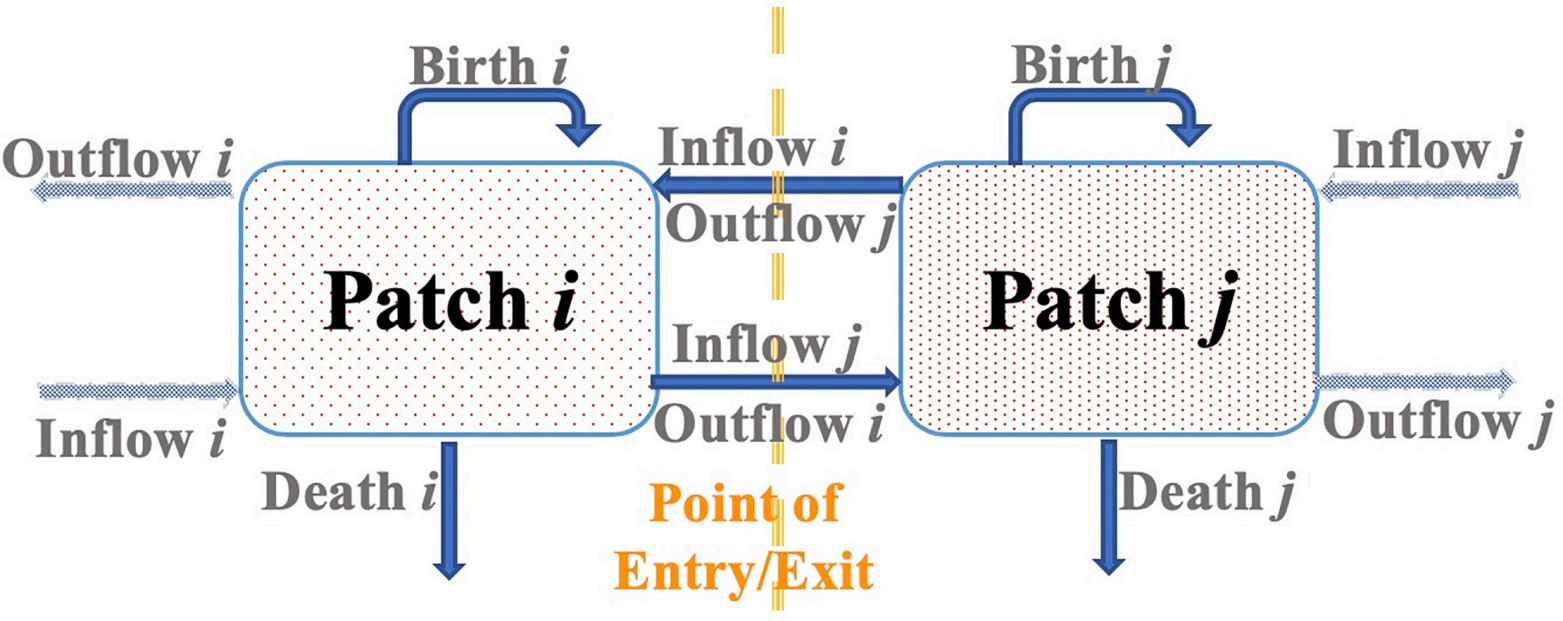
Demonstration of pathogen population dynamics in patches. Patch can be human host, zoonotic host, vector, or a piece of homogeneous environment that provides dwelling and resources for pathogen population. Each patch has four standardized pathogen population change mechanisms: birth, death, inflow, and outflow. A patch does not necessarily need to have all four of them.

The basic building block (i.e., module) of this pathogen population dynamics modeling framework is a patch *P*_*i*_. This framework has a total of four standardized mechanisms of pathogen population size change (Fig 1): inflow from any other patches, outflow to any other patches, birth (replication), and death (decay), regardless of patch type (e.g., human host, zoonotic host, vector, or environment, Eq 1). Depending on patch type and specific disease to model, each patch does not necessarily need to have all four mechanisms. For instance, many pathogens can enter the dormant state in harsh environment [39], therefore there will be no birth or death in that patch during the dormancy period. Patches are interconnected via pathogen inflow and/or outflow. Some patches may only have inflow (known as “sink”) or outflow (known as “source”) in source-sink dynamics [40]. By exploring various possibilities of connecting these modules, our modeling framework is fully modular and extensive in design.

In addition, pathogen death can be natural and/or due to medical intervention (e.g., using antibiotics for bacteria, antiviral treatment for viruses, and vaccination to trigger the host’s own immune responses). These four mechanisms are sufficiently captured by the discrete-time single population patch dynamics model shown in Eq 1. Current pathogen population size in patch *i* at time *t*+Δ*t* is the population size at previous time *t*, plus pathogen inflow from all other patches, minus pathogen out flow to all other patches, plus pathogen birth (replication) on patch *i*, and minus pathogen death (decay, both natural or due to medical intervention).

$$P_{i(t+\Delta t)}=P_{it}+\sum P_{ji\_inflow}-\sum P_{ij_{outflow}}+P_{i_{birth}}-P_{i_{death}}$$ (1)

The continuous version of this model can be derived as the following ordinary differential equation (ODE) system in Eq 2:

$$\frac{dP_{i}}{dt}=\sum_{j=1,j\neq i}^{N}\mu_{ji}P_{j}-\sum_{j=1,j\neq i}^{N}\mu_{ij}P_{i}+\beta_{i}P_{i}-\delta_{i}P_{i}$$ (2)

Where *μ_ji_* and *μ_ij_* are pathogen transmission rates from patch *j* to patch *i*, and from *i* to patch *j*, respectively. *β_i_* is pathogen birth rate in patch *i*, and *δ_i_* is the death rate in patch *i*. Environmental heterogeneity can be modeled as multiple smaller, partially connected, and homogeneous environment patches.

### Model extensions: Nonlinear terms, multi-pathogen interactions, cross-scale, and multi-transmission pathways

In the generalized model (Eq 1), the patch resource is assumed as infinity. Since this modeling framework tracks pathogen population dynamics in each patch, a finite amount of resource can be a major limiting factor for pathogen population. Therefore, Eq 2 will be rewritten to include a finite amount of resources in that patch (Eq 3) where *K*_*i*_ is the resource available in patch *i*.:

$$\frac{dP_{i}}{dt}=\sum_{j=1,j\neq i}^{N}\mu_{ji}P_{j}-\sum_{j=1,j\neq i}^{N}\mu_{ij}P_{i}+\beta_{i}P_{i}\left(1-\frac{P_{i}}{K_{i}}\right)-\delta_{i}P_{i}$$ (3)

The growth term is modeled as a logistic growth for general demonstration purposes. Nevertheless, it can be replaced by other more realistic terms for specific case studies such as a Gompertz function [41] based on user’s decision.

A special case would be pure transmitting without any pathogen replication and death in the patch (i.e., effective birth rate equals zero in any patch), shown in Eq 4:

$$\frac{dP_{i}}{dt}=\sum_{j=1,j\neq i}^{N}\mu_{ji}P_{j}-\sum_{j=1,j\neq i}^{N}\mu_{ij}P_{i}$$ (4)

An important application of such pure transmission model would be to investigate the risk of healthcare associated infections (HAIs), where multiple medical devices and surfaces could be contaminated by HAI pathogens. We have shown in our previous study that the system equilibrium can be derived analytically [42]. Therefore, we will be able to assess the equilibrium distributions of HAI pathogens among multiple environments within a healthcare facility, and identify the “high-risk” areas/devices based on pathogen population sizes on them.

As such, the original version of the modeling framework (Eq 2) can be readily extended to incorporate more realistic between-host and within-host processes. In Eq 2, pathogen death rate and birth rate, or collectively the effective birth rate, is modeled as a constant coefficient *β_i_*. Nevertheless, this coefficient can be time and/or patch dependent to include realistic interaction between the patch and pathogen subpopulations. This modeling framework is actually an agent-based model (ABM) from the host’s perspective [36, 37]. Therefore, we are able to model the effective birth rate of pathogen subpopulation at specific host *H*_*i*_ at given time *t* as a function *f* of the host’s risk factors, e.g., clinical, epidemiological, demographic risk factors such as treatment status, vaccination status, immune system level, age, gender, ethnicity, etc., such that *β_it_* = *f*(*H_it_*). Consequently, the immune response between pathogen and host, the key factor that determines the success of pathogen invasion, can be modeled by this framework as well. In addition, this model can also handle host demographics as well. For example, death of the host due to infection can be modeled as removal of the specific host patch, thus eliminating the residing pathogen subpopulation within the host patch.

Similarly, pathogen inflow and outflow coefficients are able to incorporate various external variables: *μ_ijt_* = *g*_1_(*H_it_, H_jt_*) and *μ_jit_* = *g*_2_(*H_it_, H_jt_*). For example, many pathogens are transferred through host-host contact, therefore, our previous research showed that the inflow and outflow coefficients can be modeled as a function of the contact structure between the two interacting hosts [43, 44]. With the help of more advanced monitoring systems, we are able to infer and construct the entire contact network among all individuals in the population, which give a more accurate real-time parameterization. In summary, although the foundation of this modeling framework is constructed with linear terms, it is feasible to replace these terms with more specific user-defined functions to reflect more realistic scenarios.

Our basic module builds upon patch dynamics of a single species (or serotype) of pathogen, as shown in Eqs 1 through 4. However, interspecific interaction is ubiquitous from the pathogen’s perspective, as these pathogens are part of the host microbiome or in the environment. For demonstration purposes, consider a patch having two interacting pathogens *P*_*i1*_ and *P*_*i2*_. From an ecological perspective, microbial interaction, like any ecological interaction, could be competitive (with the extreme case of competitive exclusion, seen in Fleming’s discovery of Penicillin), commensal, predatory, and mutually beneficial. The last one, mutualism among pathogens, can be dangerous to hosts (patients). Two or more pathogens which may or may not necessarily invade the same host at the same time can synergistically take down host’s immune system for co-infection (e.g., co-infection of HIV and syphilis, HIV and tuberculosis, hookworm and malaria, multiple arboviruses, and different serotypes of the same pathogen [45–47]). The system equation for each patch *P* in this scenario will be modeled as follows (assuming two pathogen species/serotypes 1 and 2 coexist and interact in each patch) in Eqs 5.1 and 5.2:

$$\frac{dP_{i1}}{dt}=\sum_{j=1,j\neq i}^{N}\mu_{j1i1}P_{j1}-\sum_{j=1,j\neq i}^{N}\mu_{i1j1}P_{i1}+\beta_{i1}P_{i1}\left(1-\frac{P_{i1}+P_{i2}}{K_{i}}\right)-\delta_{i1}P_{i1}+\alpha_{1}P_{i1}P_{i2}$$ (5.1)

$$\frac{dP_{i2}}{dt}=\sum_{j=1,j\neq i}^{N}\mu_{j2i2}P_{j2}-\sum_{j=1,j\neq i}^{N}\mu_{i2j2}P_{i2}+\beta_{i2}P_{i2}\left(1-\frac{P_{i1}+P_{i2}}{K_{i}}\right)-\delta_{i2}P_{i2}+\alpha_{2}P_{i1}P_{i2}$$ (5.2)

Note that the interaction term α_1_ and α_2_ can be positive, negative, or zero depending on the two pathogens’ actual ecological interactions in a specific patch. Even when two pathogens are mutualistic (i.e., α_1_ and α_2_ being both positive), they still have to share and compete for a limited amount of resources in the same patch. Therefore, competition for a finite amount of resources is still valid in addition to their mutualistic interaction.

Similar to a metapopulation model, this modeling framework also has the ability to scale up (e.g., tracking pathogen population dynamics in several host populations, different villages, or cities) or even scale down (e.g., tracking pathogen movement among different organs/systems within a host’s body). Additionally, we propose that our novel modeling framework can also track and model virulence plasmid exchange and replication among a bacteria community. In this scenario, the concept of “pathogen” is revolutionized: the pathogenic bacteria (originally the pathogen) is just a temporary or permanent “patch” (dwelling) of the virulence plasmid. For example, pathogenic serotypes of *Escherichia coli* (*E*. *coli*), such as enterotoxigenic, enteroinvasive, enteropathogenic, and enterohemorrhagic *E*. *coli*, all of which have and can transfer virulence plasmids among them [48].

In summary, because host (both human and zoonotic), environment, and vector are all modeled as patches of pathogens with standardized pathogen population size change mechanisms, this modeling framework can naturally handle disease transmission system with multiple transmission pathways: from environment as well as via multiple species of vectors.

### Distinctions of our model from the SIR-type model

The structure of our pathogen patch dynamics model resembles SIR-type model, such that they are both expressed as ODE systems [10]. However, there are several important distinctions. First of all, SIR-type model focuses on host epidemiological status. Pathogen, one of the three major components in epidemiological triad [26], is modeled implicitly.

Second, SIR-type model generally does not differentiate between-host transmission and within-host infection [15]. Our patch dynamics model, by comparison, explicitly models between-host pathogen movement (transmission: inflow and outflow between patches) and within-host dynamics (infection: replication). Currently, while cross-scale models also aim to incorporate both between- and within-host dynamics, these approaches are usually from host’s level as well [27–31].

Third, in SIR-type model, each compartment (e.g., *S*, *I*, and *R*) needs to be specifically formulated and there is no universal way of modeling all compartments consistently [16]. In our pathogen patch dynamics model, each patch, regardless of human host, zoonotic host, vector, or environment, has a total of four standardized mechanisms of population size change (inflow, outflow, birth, and death). Therefore, we can use one universal equation (Eq 2) to represent any kind of patch dynamics in a consistent way instead of expressing it as an array of different ODEs. This is a particular advantage of our modeling framework when dealing with environments, as there is no consensus on how to model environment in SIR-type model.

### Relationship with SIR-type model

Although SIR-type models are considered as host population level model, classification of host’s epidemiological status still needs to be all the way down to individual host’s clinical diagnosis. For example, *I* represents the collection of all identified infected hosts. We suggest that such classification is in fact a cross-sectional and discretized view of the continuous dynamics of pathogen population in hosts. As stated earlier, the original Kermack-McKendrick model was developed almost a century ago [1–7] when diagnosis was largely based upon superficial symptoms. Since then, clinical sciences have been vastly improved. Diagnosis is now based on much more concrete and accurate molecular, biochemical, and biological evidences in addition to symptoms. Living in era of Big Data, we have abundant data available to describe the longitudinal and continuous pathogen dynamics within a single host [34]. Now we know that some pathogens, for example Ebola virus, have a very rapid disease progression after initial exposure to the pathogen. Therefore, in a host-level SIR-type model, the incubation period would be neglected by assuming that susceptible hosts become infected almost immediately after exposure. Consequently, one would model Ebola as an SIR model.

**Fig 2:**
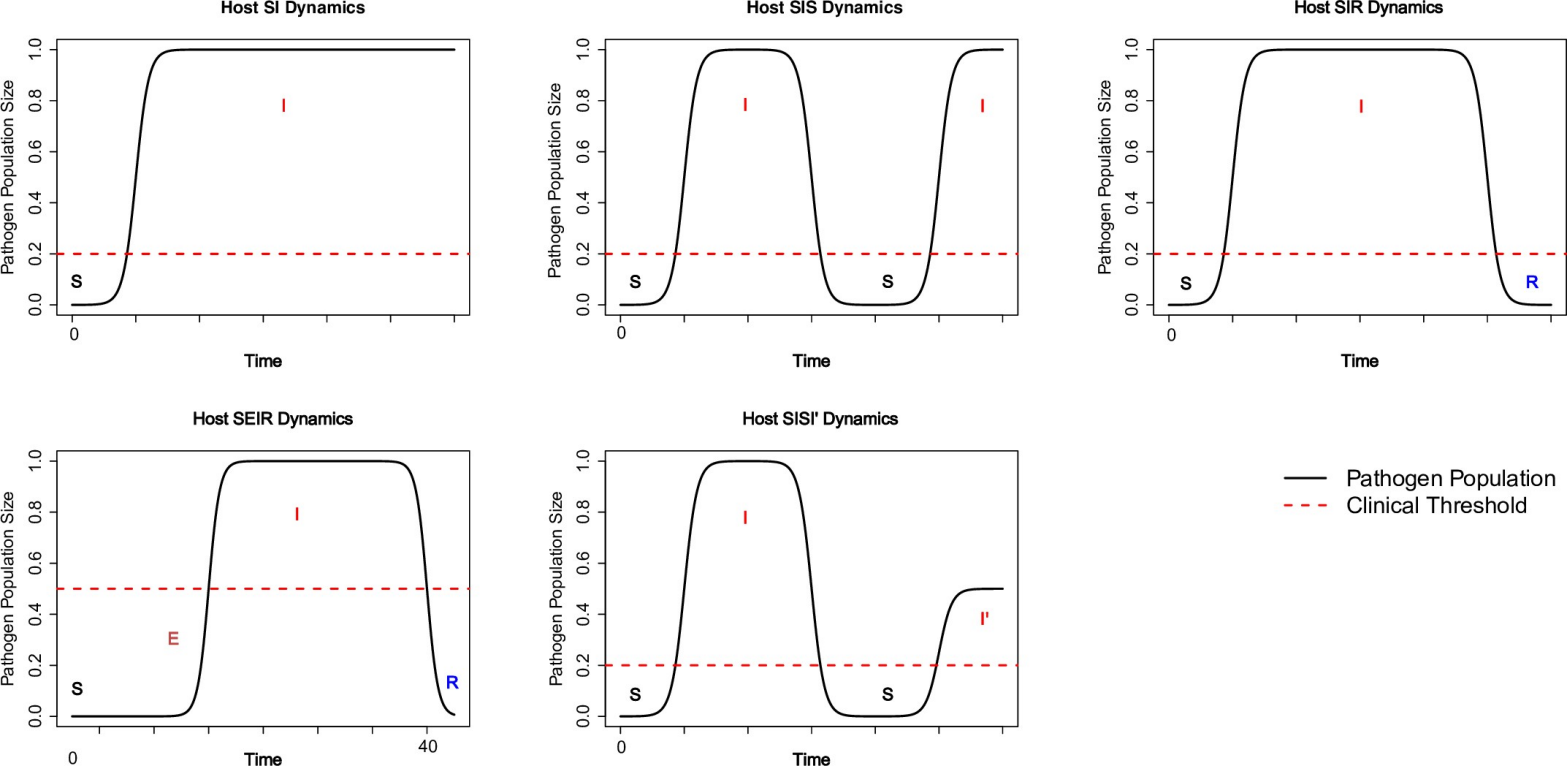
Relationships between pathogen population dynamics and individual host’s epidemiological status changes. The within-host pathogen birth (replication), which overtakes host’s immune response, indicates an infection and changes host’s status from susceptible (S) to infected (I) in commonly used SIR-type model. Sometimes it takes a while for pathogen population size to increase substantially to make the host susceptible and during which the host is considered as exposed (E) status.

In contrast, other pathogens such as HIV may have a much longer incubation period during which the host remains asymptomatic/subclinical, and it could be modeled as an SEIR model in which *E* represents an exposed but not infectious state. Hosts gaining partial immunity against pathogen will be modeled as SISI’, where *I’* represents less intense successive infection after the initial infection. An illustration of the relationship between patch dynamics and host epidemiological status in different scenarios is provided in Fig 2. Note that each host is individually tracked, therefore each host’s threshold of showing clinical symptoms can be different, depending on the host’s own physiological and pathological condition (e.g., vaccination or comorbidity). This is another advantage of our modeling framework over the SIR-type model. Our novel framework is an ABM from hosts’ perspective which naturally considers heterogeneity among hosts, resonating with the concept of “precision medicine”.

A special case of our patch dynamics modeling framework is an occupancy model. In an occupancy model, a patch is either occupied by the pathogen (1, corresponding to *I* status in SIR-type model) or not occupied (0, corresponding to *S* or *R* status, depending on that specific host’s previous history of infection). This is indeed the ABM version of the SIR-type model because of the discretization (dichotomy of patch occupancy) of a continuous pathogen quantity. For pathogens that are difficult to quantify but relatively easy to detect (e.g., SARS-CoV-2 in wastewater), this occupancy model (or equivalently the ABM version of SIR-type model) will be preferred [33]. If the occupancy model is exclusively for multiple fixed environments, then it can also be regarded as a cellular automata (CA) model [49].

**Fig 3:**
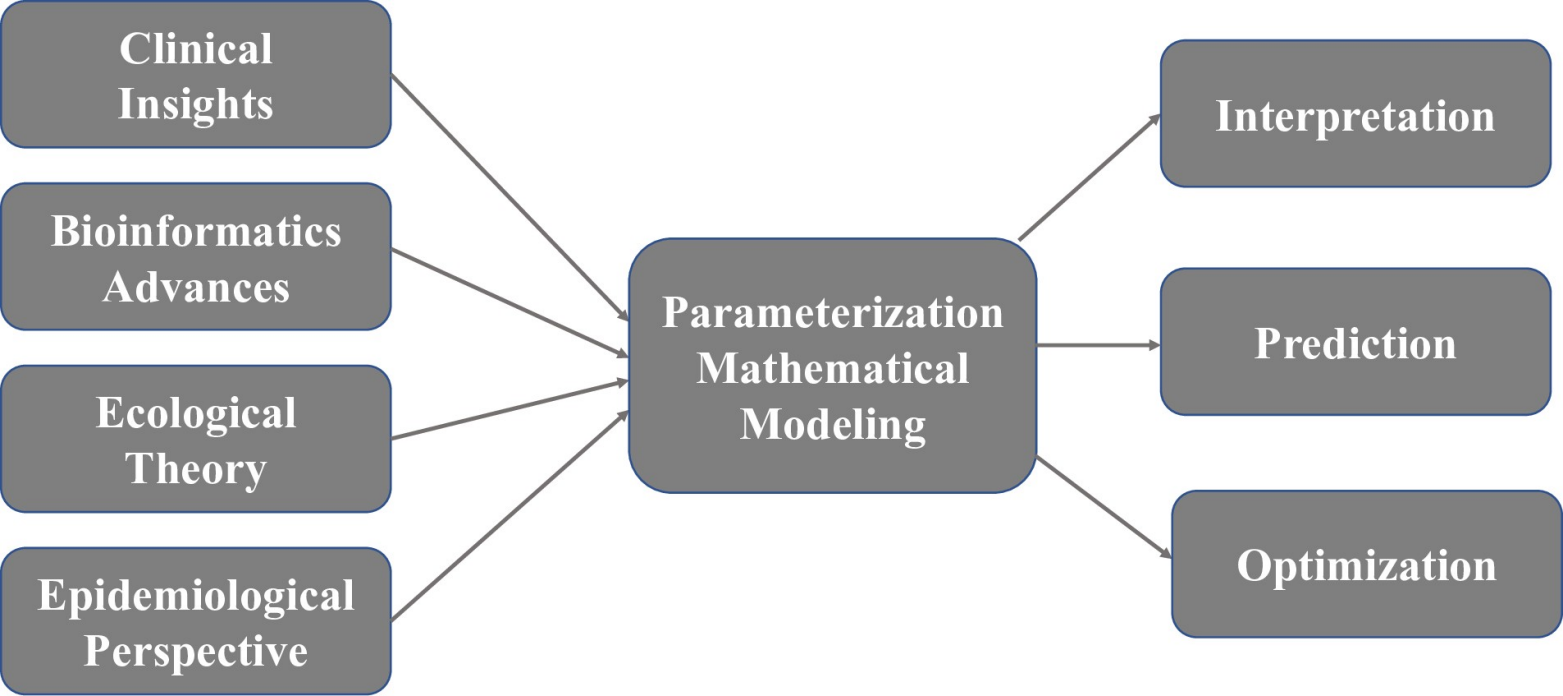
Schematic view of model foundations, inputs and uses. This is a demonstration of potential model inputs and applications. It is not an exhaustive list. The actual inputs can be determined by the specific study system and data availability.

So far, we have established the alternative methodology of modeling infectious disease through pathogen patch dynamics, while still incorporating host and environment explicitly. It has the potential of interpreting existing infectious disease phenomenon, predicting future outbreaks more accurately, and guiding optimal intervention strategies for infectious disease transmission. A schematic view of how our modeling framework works is provided in Fig 3. Next, we provide two case studies to demonstrate the flexibility and feasibility of our modeling framework. Each case study tackles a challenging scenario for SIR-type model and serves as a proof-of-concept theoretical study of our modeling framework.

### Case study 1: Role of super-contacter in STD transmission

The first case study focuses on sexually transmitted diseases (STD) where the underlying sexual contact network is far from homogeneous mixing (i.e., there might be a few active super-contacters and remaining normal contacters [50, 51]). STD transmission relies on the underlying contact network. However, it was unclear whether super-contacters are responsible for disproportionately large proportion of transmitting STDs. In addition, people may wonder whether having a monogamous relationship is sufficient to protect against STD. These questions are difficult to answer with SIR-type model because the underlying assumption of homogeneous mixing (i.e., each host has an equal probability of contacting any other hosts), which is generally far from reality in STD transmission [18, 21].

Here, we simulate a closed population of five hosts (N = 5) whose contact network is known. Host 1 (H1) will be the “super-contacter” who has a sexual relationship with all other members in the population. Host 4 (H4) has a unidirectional monogamous relationship with H1. Initial condition (IC) of pathogen amount in each host and parameters for this simulation are provided in S1 Table. The parameters and initial conditions are based on responses from deidentified participants on contact structure and their clinical results.

### Case study 2: How superinfection in healthcare associated infection works?

In this second case study, we aim to illustrate and explain the super-infection phenomenon in healthcare associated infection (HAI). Here we consider two pathogens that infect the same host population indirectly through contaminated environment. An SIR-type model could handle co-infection by further dividing host population into subpopulations of different epidemiological states of each pathogen. However, our pathogen patch dynamics model provides a much more natural and effective way to model coinfection and superinfection as interspecific interaction (e.g., competitive, commensal, and mutualistic in the same patch).

An example would be facultative bacterium *Clostridium difficile* initially infects a host who has been administerered broad-spectrum antibiotics to treat a previous infection, therefore disrupting the microbiome and leaving room in the host “patch” for facultative *C*. *difficile* [52]. For demonstration purposes, we simulate two pathogens, one less pathogenic and the other much more pathogenic (e.g., *C*. *difficile*). There are two hosts, neither of whom have had *C*. *difficile* infection before. *C*. *difficile* survives inside healthcare facility without birth or death (i.e., in dormant state). One of the hosts used antibiotics to substantially reduce less-pathogenic pathogen’s population which causes *C*. *difficile* to enter and replicate rapidly within the first host’s body, shed to the environment, caused infection of next host, and initialize an outbreak in the facility. The initial condition (IC) of pathogen amount in each host as well as environment, and parameters for this case study are provided in S2 Table. The parameters in this case study are hypothetical and for demonstration of feasibility purpose only.

## Results

### Generalized modeling framework and system equilibrium

For each patch, if $\frac{dP_{i}}{dt}=0$ then the pathogen population on this patch is in dynamic equilibrium (steady state), therefore its population size does not change. If $\frac{dP_{i}}{dt}>0$ then the pathogen population size increases. Assuming *P*_*i*_ is in host, this will eventually result in infection (i.e., multiplication of pathogens within the host). Some pathogens, for example Ebola virus that causes hemorrhagic fever, have very rapid progression after initial exposure and have very high infectivity, pathogenicity, and virulence. Other pathogens may slowly reach the threshold when clinical symptoms emerge (e.g., cervix cancer develops years after initial HPV infection). The relationship between patch dynamics of pathogens (especially in host) and SIR-type compartment model can be found in Fig 2. Otherwise, if $\frac{dP_{i}}{dt}<0$ then the pathogen population size will eventually decline to zero, which is the trivial yet stable system equilibrium. From the host’s perspective, the pathogens have failed to colonize the host, which is a desirable outcome from a disease control perspective.

### Model parameterization, complexity, and feasibility

For a direct host-host transmission-infection system with *N* hosts (patches), there will be a maximum of *N*^*2*^*-N* parameters for inflow, *N*^*2*^*-N* parameters for outflow, *N* for death, and another *N* for birth. Therefore, in theory there will be a total of *2N*^*2*^ parameters needed if every host is in contact with each other, which forms a complete network. However, due to the Pareto principle (commonly known as the 20/80 rule), the underlying contact network among hosts should be far less than a fully connected network [53–56]. Such network is known as scale-free network and degree distribution approximates power law [57]. In contrast, SIR-type models, which usually assumes random mixing of hosts, do not represent the power law distribution of host contact well.

If we could assume *u*_*ij*_ *= u*_*ji*_ (symmetry of transmission regardless of direction), then the number of parameters is much fewer. In addition, if we could further estimate an effective birth rate (combined effect of both birth and death in the same patch), then the number of parameters can be even fewer, making this modeling framework more feasible to deal with.

For indirect host-environment transmission system where hosts only acquire and shed pathogen from and to the environment (no direct host-host interactions for disease transmission), the number of parameters needed is roughly 4*N*, assuming *N>>*1. Since our modeling framework is most often a linear system, it can be simulated and solved (e.g., deriving system equilibria) more efficiently by a wide range of algorithms and packages, comparing to nonlinear SIR-type models.

Parameters in this modeling framework can be estimated from a wide range of ever-increasing sources, summarized concisely in Table 1. These are just selected examples of potential sources of parameterization. As science and technology keep evolving, it is expected more sources will be available and accessible to accurately estimate parameters via newly developed analytical techniques such as machine learning. Therefore, we conclude that our modeling framework can be used to interpret existing infectious disease epidemiological phenomena, predict possible spatio-temporal transmission patterns, and optimize potential intervention strategies (Fig 3).

**Table 1:** Parameters and corresponding processes in the pathogen patch dynamics model.

Parameter	Interpretation	Parameterization	Notes
μ_ij_, μ_ji_	Inflow and outflow rate of pathogen between patches	Genomic data [58], contact structure between host and environment or among hosts from real-time location system (RTLS), electronic health records (EHR).	Directional (from patch *i* to *j* or from *j* to *i*). Note that *i*, and *j* indicate patches while in parameter α, *m* and *n* indicate pathogen species.
β_i_	Birth rate in patch *i*	*in vivo* and/or *in vitro* studies and existing literature.	Can be consolidated with death rate to form a single effective birth rate
δ_i_	Death rate in patch *i*	*in vivo* and/or *in vitro* study, existing literature, etc.	Can be both natural death and intervention-related death (e.g., treatment with antibiotics, immunization reaction from vaccination, etc.)
α_mn_	Pathogen interaction rate between pathogen species *m* and *n* (when applicable)	microbiology study and existing literature.	Can be positive, negative, or zero depending on the actual interaction (e.g., mutualism, competition, or commensalism.)

### Case study 1: Role of super-contacter in STD transmission

**Fig 4:**
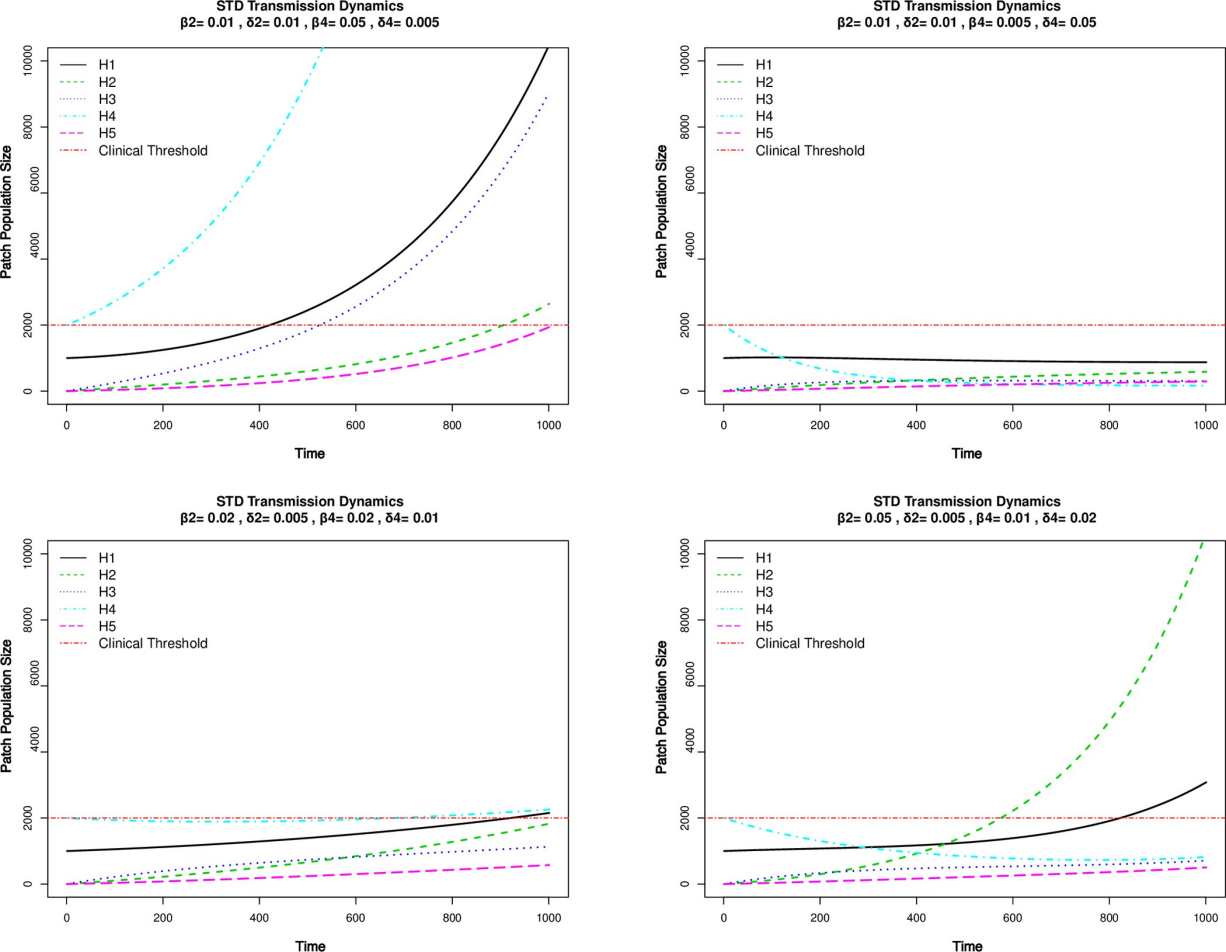
STD dynamics in hosts based on different pathogen birth/death rates. A total of five hosts (H1-H5) are simulated with varying pathogen population demographic parameters (birth rate *β_i_, i* = 1…5) and death rate *δ_i_, i* = 1…5) showing different susceptibility of the hosts to HIV infection. Transmission rates are provided in S1 Table.

An illustration of different pathogen patch dynamics among these hosts in four hypothetical conditions is provided in Fig 4. For the specific parameter and initial condition (IC) settings, H1 does not qualify super-spreader. Panel 1 (top left) shows that H4 shows clinical symptoms the earliest and has the most pathogens on him/her, mostly due to the largest pathogen replication rate among all hosts. In addition, H2 (green line) in condition 1 eventually reaches clinical threshold and has a very long incubation period. Similar findings are shown in panel 3 (bottom left) for H1 and H4. Most disturbingly, in the last condition (panel 4, bottom right), the monogamous H2 who has never had STD before, is clinically diagnosed first and has the largest amount of pathogen eventually. These results show that in addition to understanding the underlying contact network, we need to have more precise clinical and epidemiological information about these individuals in order to make accurate predictions of disease dynamics.

This case study also demonstrates the important and novel concept of U = U (undetectable equals un-transmissible) especially in HIV intervention [59]. Although currently there is no cure for HIV-positive individuals, they can take antiviral treatment (ART) regularly to suppress the viral load below detectable amount (e.g., <200 copies of viral RNA/mL in blood, commonly known as “suppressed” status). These hosts are still carriers of HIV virus, but transmission does not typically occur because of extremely small number of pathogens present. This is another example of how current clinical insights are difficult to represent in SIR-type model. Demonstration of U = U is depicted in Fig 4 panel 2 (top right) and large death rates of HIV can be the effect of antiviral treatment. Note that as a demonstration, we ignore the initial phase of rapid virus replication within the first few days of HIV infection. This can be easily adjusted when necessary.

An important application of our modeling framework is to evaluate potential prevention and intervention strategies. In current U = U practice, HIV patient is given regular antiviral treatment [59]. As discussed earlier, our modeling framework is also an agent-based model (ABM) from host perspective, therefore pathogen death rate δ in host *i* at any given time *t* can be further expressed as two additive components: a constant natural death rate δ_0it_ and a variable death rate δ_1it_. The variable death rate is due to periodic administration of ART every *T*-days and when ART is administrated, it will reduce HIV pathogen at a rate of δ_ART_ (Eq 6):

$$\delta_{it}=\delta_{0it}+\delta_{1it},\;\mathrm{where}$$

$$\delta_{1it}=\left\{ \begin{array}{c} \delta_{ART},\;t=nT,n=1,2,3,\ldots ,N.\\ 0,\;t\neq nT \end{array}\right.$$ (6)

The variable pathogen death rate δ is modeled as a form of impulse response function, and more realistic impulse response functions can be adopted. This will help us optimize administration of ART for each specific HIV patient (e.g., identifying an optimal combination of interval or dose, that will result in the suppressed state of the patient). Future studies can be based upon other factors such as financial concerns (e.g., cost-effectiveness analysis) to perform optimal control at individual HIV patient level. Optimization is an important application of precision medicine, as well as one of the major outputs of this modeling framework in addition to making accurate interpretation and prediction (Fig 3).

### Case study 2: How superinfection in healthcare associated infection works?

**Fig 5:**
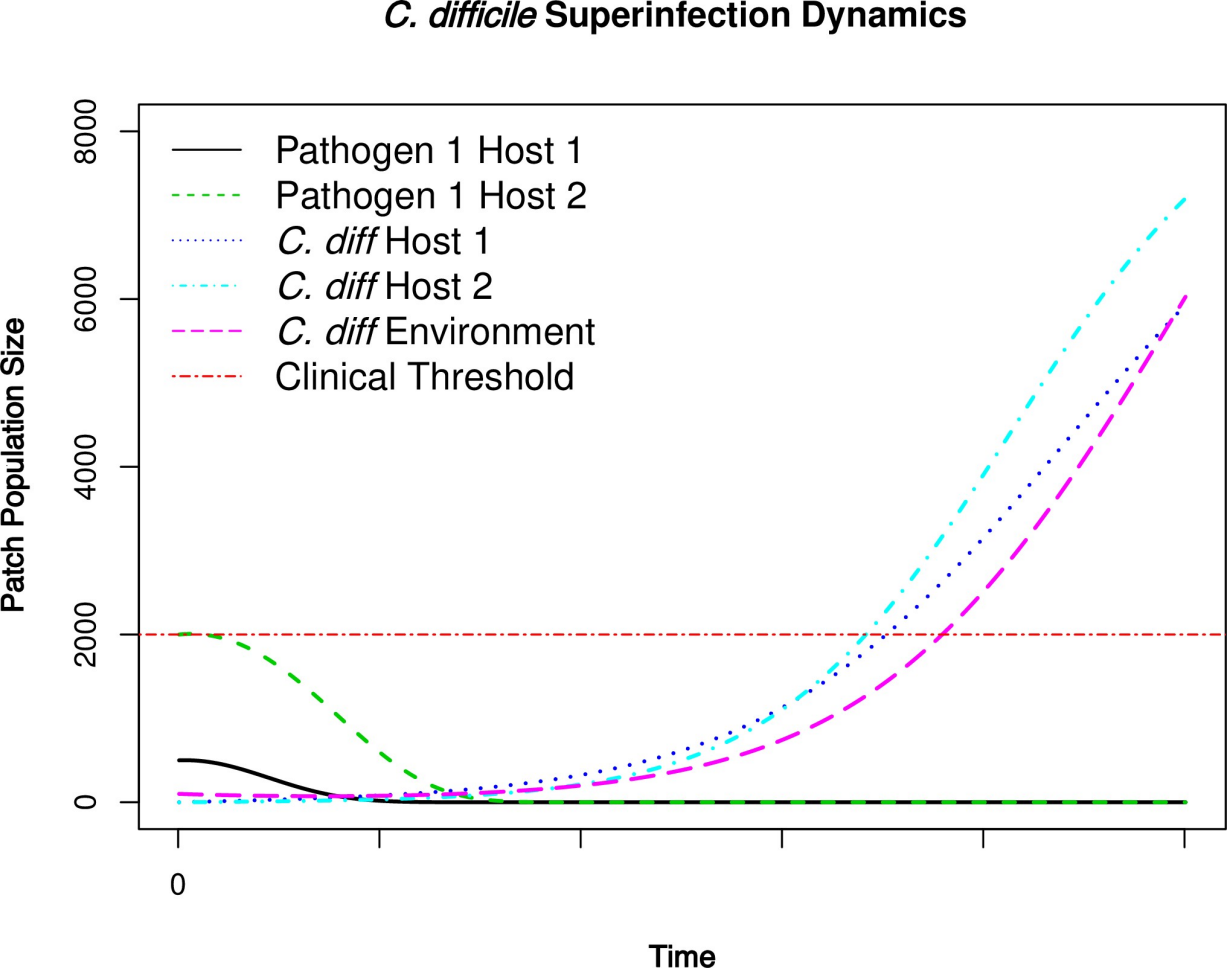
HAI dynamics of two competing pathogens and superinfection with *C*. *difficile*. Initial condition and model parameters are provided in S2 Table.

The process of super-infection is characterized as two competing species (one of which being the “superbug” *C*. *difficile*) in host patch and their patch dynamics is captured in Fig 5. In this case, environment patch serves as the reservoir for *C*. *difficile*. In conservation ecology, this is known as the rescue effect, where subpopulation in one or a few patches can be used to “rescue” the main population via re-introduction. Nevertheless, from a public health perspective, such rescue effect actually proposes challenges to superinfection prevention. Simulation results show that less pathogenic pathogen 1 in H1 declines as the result of broad-spectrum antibiotics use (black line). However, *C*. *difficile* takes over H1 (dark blue line) and H1 sheds the *C*. *difficile* pathogens back to the environment. This makes *C*. *difficile* amount in environment increase as well, and causes further *C*. *difficile* infection in H2 (light blue line). As *C*. *difficile* population size increases in H2, it simultaneously inhibits pathogen 1’s population in H2 (green line). The final result is the competitive exclusion of pathogen 1 in both hosts.

Since *C*. *difficile* is a type of facultative pathogen and can survive in harsh environments in a dormant state, we need to make sure that we reduce environmental pathogen to a very low population size before *C*. *difficile* has any chance of facultative infection. More importantly, we need to be very cautious when applying antibiotics that may disrupt the microbiome in patients’ bodies, which gives *C*. *difficile* an opportunity of infestation and superinfection.

## Discussion

In this study, we provide a unified methodological framework for modeling infectious disease dynamics with rigorous ecological and epidemiological foundations. A human host, a zoonotic host, a piece of homogenous environment, and a vector are all examples of patches, the dwellings for pathogen populations that live in. A patch is the standard module in this modeling framework and patch dynamics of pathogen can be described in a universal way with four pathogen population size change mechanisms: birth, death, inflow, and outflow. The relatively simple mathematical expression and universal adaptability enables this modeling framework to be adopted to investigate much more complicated scenarios of transmission dynamics. For example, transmission with non-homogeneous mixing (contact structure), co-infection with multiple interacting pathogens (or serotypes/strains), transmission with multiple transmission pathways, all of which are challenging to SIR-type models [12–15], can be formulated by connecting the interacting patches. Therefore, it is our firm belief that in the Big Data era when data-driven machine learning models are making tremendous progress in predicting infectious disease outbreaks [60, 61], relatively simple and interpretable mechanistic models (e.g., our patch dynamics model or SIR-type models) are still relevant and useful. In fact, with larger amounts of easily accessible data, machine learning methods can be applied to parameterize for the mechanistic model (as shown in Fig 3) and complement each other to characterize infectious disease dynamics more accurately [34]. In addition, current COVID-19 pandemic proposes substantial challenge to the common SIR-type modeling paradigm, especially how unknown proportion and contribution of asymptomatic patients can be adequately and accurately incorporated in the models. We will further develop a pathogen-oriented COVID-19 transmission model based on the modeling framework in this study. Symptomatic and asymptomatic patients are two types of patches differing in host susceptibility and infectivity. More clinical and epidemiological inputs can also be included in our novel modeling approach (Fig 3). We believe our modeling framework can handle more complicated epidemic systems such as current COVID-19 pandemic [62].

Another important extension of this modeling framework is to further incorporate genomic or other -omics data. The cost and accuracy of next-generation sequencing (NGS) has enabled highly granular characterization of different strains of pathogens [28, 29]. Therefore, strain-specific interactions within the same patch, for instance, co-infection and inter-specific competition of more than one strain in the same host (e.g., superinfection of different strains of dengue virus [63]). Because this modeling framework builds on solid ecological foundations, even more complicated scenarios, such as cross-scale modeling of more than one host or vector, can be investigated. One example will be the hierarchical food web interactions that occur to pathogenic *Escherichia coli* in the environment. Nematodes in the soil prey on or carry *E*.*coli*, various fly species mechanically carry nematodes and further spread the nematodes containing *E*.*coli* to hosts such as farm animals or humans. All these complicated interactions and their associated process parameters could be identified and quantified with the help of NGS. Therefore, our modeling framework will provide a more comprehensive and accurate depiction of pathogen transmission in the ecosystem.

In addition to the advantage of extensibility of our modeling framework, it is also a scalable system. “Patch”, defined as a relatively homogeneous dwelling, can be larger (scaling up) or smaller (scaling down) [38]. Patch dynamics of pathogens is considered invariable of the patch’s actual physical size (i.e., each patch has exactly the same four population size change mechanisms). Scaling up, our modeling framework can handle pathogen flow among villages/communities in various suburban or urban setting, as seen in metapopulation models [64–67]. Scaling down, even within a host, pathogens may move around and invade specific organs/systems [68]. Such within-host dynamics can also be readily captured by our patch dynamics model to investigate pathogen-immune system responses.

If we can scale down even further into a pathogenic bacterium cell, for instance, *E*. *coli*, we can consider virulence plasmid as the actual “pathogen” while the bacterium cell is nothing but a “patch” of the plasmid. Non-pathogenic serotypes of *E*. *coli* can acquire a virulence plasmid, many of which can cause antimicrobial resistance, via direct bacterium-bacterium conjugation (similar to direct host-host transmission) or indirect transformation from environment [48]. Therefore, plasmid’s patch dynamics can be tracked by this modeling framework, and we are able to use it to evaluate and predict potential outbreak of pathogenic serotypes and risk of antimicrobial resistance.

This framework provides a promising and feasible alternative solution to model infectious disease dynamics. With the advancement of modern medicine, health sciences, technology, and the unprecedented large amount of data they produce, model parameterization is made possible. Genomics data from hosts, vectors, and pathogens, behavior information of hosts, information in electronic health records that provides a continuous viewpoint of individual’s health, all contributes to more accurate parameterization of this model. We suggest that right now, this modeling framework would work the best with a small, semi-closed, and closely monitored human host population. Nevertheless, with newer, more available and more accurate data sources, this modeling framework should also be able to handle disease transmission in relatively large host population. As discussed earlier, this scalable framework enables it to operate at larger scale (which generally encompasses larger host population) to indirectly handle larger host population [43, 44, 62].

In conclusion, we provide a simple, universal, versatile, yet powerful mathematical modeling framework to model infectious disease dynamics based on pathogen patch dynamics. Future studies can adopt this modeling framework to investigate a wide array of emerging and critical issues related to infectious diseases, generate new insights, and develop more effective intervention strategies.

## Supporting information

S1 Table Parameters and initial conditions to simulate STD transmission in case study 1. (DOCX)

S2 Table Parameters and initial conditions to simulate HAI transmission in case study 2. (DOCX)

## Abbreviations

ABM: agent-based model
ART: antiviral treatment (for HIV)
CA: cellular automata
DENV: dengue virus
E: environment
H: host
HAI: healthcare associated infection (or hospital acquired infection)
HIV: human immunodeficiency virus
HPV: human papillomavirus
IC: initial condition of the ODE
ODE: ordinary differential equation
P: patch
SIR: susceptible, infected (or infectious, infective), and recovered
STD: sexually-transmitted disease
V: vector
VBD: vector-borne disease
