# Supplementary material for: Patch dynamics modeling framework from pathogens’ perspective: Unified and standardized approach for complicated epidemic systems
Source: PLoS One. 2020 Oct 15;15(10):e0238186. doi: 10.1371/journal.pone.0238186 (PMC7561140; doi:10.1371/journal.pone.0238186)
Supplement: S1 Table — (DOCX) [file pone.0238186.s001.docx]

**S1 Table. Parameters and Initial Conditions to Simulate STD Transmission in Case Study 1**

| **Transmission Rate**  **From-To** | **H1** | **H2** | **H3** | **H4** | **H5** |
| --- | --- | --- | --- | --- | --- |
| **H1** | 0 | 0.01 | 0.005 | 0.008 | 0.003 |
| **H2** | 0.01 | 0 | 0 | 0 | 0 |
| **H3** | 0.005 | 0 | 0 | 0.01 | 0.005 |
| **H4** | 0.008 | 0 | 0.01 | 0 | 0 |
| **H5** | 0.003 | 0 | 0.005 | 0 | 0 |

|  | **Birth Rate** | **Death Rate** | **Initial Condition (IC)** |
| --- | --- | --- | --- |
| **H1** | 0.02 | 0.005 | 1000 |
| **H2** | 0.01/0.01/0.02/0.05 | 0.01/0.01/0.005/0.005 | 0 |
| **H3** | 0.005 | 0.01 | 0 |
| **H4** | 0.05/0.005/0.02/0.01 | 0.05/0.005/0.01/0.02 | 2000 |
| **H5** | 0 | 0 | 0 |

Note: in this case study, a total of four scenarios are simulated with varying birth rates and death rates of pathogens in host H2 and H4. For demonstration purposes, the transmission rates are formed in a symmetric matrix, but it can be formed as non-symmetric matrix as well.
