# Supplementary material for: Patch dynamics modeling framework from pathogens’ perspective: Unified and standardized approach for complicated epidemic systems
Source: PLoS One. 2020 Oct 15;15(10):e0238186. doi: 10.1371/journal.pone.0238186 (PMC7561140; doi:10.1371/journal.pone.0238186)
Supplement: S2 Table — (DOCX) [file pone.0238186.s002.docx]

**S2 Table. Parameters and Initial Conditions to Simulate HAI Transmission in Case Study 2**

| **Transmission Rate**  **From-To** | **H1**  ***C. difficile*** | **H2**  ***C. difficile*** | **E** |
| --- | --- | --- | --- |
| **H1** | 0 | 0 | 0.03 |
| **H2** | 0 | 0 | 0.02 |
| **E** | 0.01 | 0.01 | 0 |

|  | **Birth Rate** | **Death Rate** | **Initial Condition (IC)** |
| --- | --- | --- | --- |
| **H1, Pathogen 1** | 0.02 | 0.01 | 500 |
| **H1, *C. difficile*** | 0.08 | 0 | 0 |
| **H2, Pathogen 1** | 0.02 | 0.01 | 2000 |
| **H2, *C. difficile*** | 0.08 | 0 | 0 |
| **E, Pathogen 1** | 0 | 0 | 0 |
| **E, *C. difficile*** | 0 | 0 | 100 |

Note: in this case study, we assume that neither less pathogenic pathogen 1 nor *C. difficile* replicate or decay in the environment (E). In addition, the inter-specific competition is only upon less pathogenic pathogen’s population but not on *C. difficile* population. The interaction coefficient is 0.005 in both H1 and H2.
